# Next-Generation Sequencing for Whole-Genome Characterization of *Weissella cibaria* UTNGt21O Strain Originated From Wild *Solanum quitoense* Lam. Fruits: An Atlas of Metabolites With Biotechnological Significance

**DOI:** 10.3389/fmicb.2021.675002

**Published:** 2021-06-07

**Authors:** Gabriela N. Tenea, Pamela Hurtado

**Affiliations:** Biofood and Nutraceutics Research and Development Group, Faculty of Engineering in Agricultural and Environmental Sciences, Technical University of the North, Ibarra, Ecuador

**Keywords:** whole-genome sequencing, folates, penicillin acylase, *Weissella cibaria*, antimicrobial activity, *Staphylococcus aureus*

## Abstract

The whole genome of *Weissella cibaria* strain UTNGt21O isolated from wild fruits of *Solanum quitoense* (naranjilla) shrub was sequenced and annotated. The similarity proportions based on the genus level, as a result of the best hits for the entire contig, were 54.84% with *Weissella*, 6.45% with *Leuconostoc*, 3.23% with *Lactococcus*, and 35.48% no match. The closest genome was *W. cibaria* SP7 (GCF_004521965.1) with 86.21% average nucleotide identity (ANI) and 3.2% alignment coverage. The genome contains 1,867 protein-coding genes, among which 1,620 were assigned with the EggNOG database. On the basis of the results, 438 proteins were classified with unknown function from which 247 new hypothetical proteins have no match in the nucleotide Basic Local Alignment Search Tool (BLASTN) database. It also contains 78 tRNAs, six copies of 5S rRNA, one copy of 16S rRNA, one copy of 23S rRNA, and one copy of tmRNA. The *W. cibaria* UTNGt21O strain harbors several genes responsible for carbohydrate metabolism, cellular process, general stress responses, cofactors, and vitamins, conferring probiotic features. A pangenome analysis indicated the presence of various strain-specific genes encoded for proteins responsible for the defense mechanisms as well as gene encoded for enzymes with biotechnological value, such as penicillin acylase and folates; thus, *W. cibaria* exhibited high genetic diversity. The genome characterization indicated the presence of a putative CRISPR-Cas array and five prophage regions and the absence of acquired antibiotic resistance genes, virulence, and pathogenic factors; thus, UTNGt21O might be considered a safe strain. Besides, the interaction between the peptide extracts from UTNGt21O and *Staphylococcus aureus* results in cell death caused by the target cell integrity loss and the release of aromatic molecules from the cytoplasm. The results indicated that *W. cibaria* UTNGt21O can be considered a beneficial strain to be further exploited for developing novel antimicrobials and probiotic products with improved technological characteristics.

## Introduction

*Weissella* is a genus of lactic acid bacteria (LAB) consisting of species formerly included in the *Leuconostoc paramesenteroides* group and commonly found in fermented foods, milk, vegetables, feces, environment, and vertebrates including humans (Du et al., 2018; Panthee et al., 2019). Several strains, mostly belonging to the species *Weissella cibaria*, *Weissella confusa*, and *Weissella paramesenteroides*, have been widely investigated for probiotic characteristics (Tanizawa et al., 2014; Xia et al., 2019; Pabari et al., 2020) and for their capacity to protect against bacterial and fungal infection (Li et al., 2017; Wang et al., 2017). Some strains showed effectiveness in maintaining host health due to their anti-toxicity, antitumor activity, and immunomodulatory properties (Hong et al., 2016; Ojekunle et al., 2017; Yu et al., 2018, 2019).

Despite the widespread importance, the genomic characterization of this genus remains of importance. Presently, according to the genome assemblies available in the public database, the genome of 20 species of *Weissella* was sequenced. Although some strains are known to act as opportunistic pathogens, a higher level of interest was shown for the bacteriocinogenic *Weissella* strains as they are producing antimicrobial metabolites such as organic acids, hydrogen peroxide, diacetyl, and bacteriocins (Kamboj et al., 2015). For example, *W. cibaria* strain CMU showed a significant antimicrobial activity against oral pathogens (Jang et al., 2016). Currently, *Weissella* spp. were isolated from hosts such as camels from arid and desert environments and showed attractive metabolic characteristics (Fhoula et al., 2018; Manberger et al., 2020). In other studies, *W. cibaria* were isolated from dairy cows in Kuwait showing probiotic features (Patrone et al., 2020). *Weissella* species were found in various fruits such as watermelon, grapes, and tomatoes exhibiting good acidification potential, antimicrobial activity, and exopolysaccharide production (Fessard and Remize, 2017). Nonetheless, their biotechnological characteristics are highly ligated to their origin.

Ecuador is a megadiverse country rich in biological materials such as luxuriant and exotic plants that might be considered a reservoir of a high content of beneficial microorganisms. In the fermentation process, some antimicrobial substances are produced by the microorganisms habituating in fruits, which can help to improve food safety via inhibition of spoilage or pathogenic microorganisms found in the same niche. Recently, there has been a growing interest in the special use of tropical fruits not only as food but also in the characterization of the microbial population associated with those fruits. In fact, the microbiota associated with wild Amazonian tropical fruits was investigated for the presence of beneficial microorganisms such as LAB (Tenea et al., 2020a). Several wild fruits were found as an important source of LAB strains. Some isolates were molecularly classified into the genera *Lactiplantibacillus* (31 isolates), formerly *Lactobacillus*; *Lactococcus* (three isolates); *Weissella* (three isolates); and *Enterococcus* (one isolate). Among them, one isolate annotated UTNGt21O, of wild naranjilla fruits (*Solanum quitoense L.*), was sequenced and taxonomically classified as *W. cibaria* (Tenea et al., 2020b). The peptide extract of the UTNGt21O strain has a bacteriolytic mode of action toward *Salmonella* and *Escherichia coli*. By employing the BAGEL 4 software (a bacteriocin genome mining tool), a putative bacteriocin having 33.4% sequence similarity to enterolysin A was found in contig 12 along with several ORFs (11) encoding unknown proteins (Tenea et al., 2020b).

The development of an efficient probiotic product and/or a novel antimicrobial agent relies on the proper characterization of the selected strain to secure the desired benefit in the target host. Genome characterization of probiotic strains will help to predict their capabilities and functionalities as well as provide information about their mode of action for further improvement and selection of a new generation of probiotics. In the light of our recent findings showing the high inhibitory potential of the UTNGt21O strain, in this study, genome characterization was performed to identify specific genetic features with promising insights in biotechnology, food science, and antimicrobials. A gene mapping was performed to identify the potential involvement of the predicted genes in different biological pathways as well as to detect genes encoding for probiotic features. The presence of CRISPR sequences, mobile genetic elements, antibiotic-resistant genes, and virulence factors was determined via genome analysis. A pangenome analysis was performed to identify core, accessory, and unique proteins as well as to search for the presence or absence of genes. Moreover, to overcome the contamination of fruit-based products by *Staphylococcus aureus*, a hazardous, antibiotic-resistant, foodborne pathogen, which causes human illnesses after consumption, in this study, we propose to evaluate the effects of the peptide extracts from the UTNGt21O strain on the cell integrity and permeability of *S. aureus* ATCC1026, as the new antimicrobials might show a particular mode of action, which will further help to control the microorganism growth in foods. Identification of new genes through whole-genome sequencing and sequence analysis will be beneficial to comprehend their functional characteristics and technological properties for further usage in different biotechnological processes.

## Materials and Methods

### Genome Sequencing, Assembly, and Gene Prediction

The *W. cibaria* UTNGt21O genome sequence and assembly were previously deposited in 2020 at the National Center for Biotechnology Information (NCBI) with BioProject ID: PRJNA639289 and has 31 contigs. The whole-genome sequencing was performed as described (Tenea et al., 2020b). To taxonomically classify the species, identify species or/and strain information, an average nucleotide identity (ANI) (Goris et al., 2007) analysis was performed with the taxon of the reference sequence (taxon ID: 137591, *W. cibaria* BC14) matched with contig 1 by Basic Local Alignment Search Tool (BLASTN) (custom assay project, Macrogen, South Korea). The following tools were used to predict each functional element: Prodigal for CDS prediction (Hyatt et al., 2010), RNAmmer for rRNA prediction^1^, ARAGORN for tRNA/tmRNA prediction^2^, Signal IP for signal leader peptide prediction^3^, and Infernal for non-coding RNA prediction (Nawrocki and Eddy, 2013). After gene prediction was completed with the Prodigal prediction of protein-coding sequences, ORFs and subsequent gene annotation were performed using the Microbial Genome Annotation Pipeline online server (Prokka v1.14.5), by which coding sequences and rRNAs and tRNAs were predicted (Seemann, 2014). Prokka conducts an analysis on the basis of ISfinder, NCBI Background Reference Gene DB, UniProtKB DB, and HMM DB. Genome map of *W. cibaria* UTNGt21O was predicted using CGView (Grant and Stothard, 2008).

### CRISPR Sequences, Prophage, Resistance Genes, Virulence Factors, and Pathogenicity Prediction

CRISPRFinder^4^ and PHAge Search Tool Enhanced Release (PHASTER)^5^ (Grissa et al., 2007; Seemann, 2014) were used to detect CRISPR, Cas sequences, truncated Cas sequences, identification, and annotation of prophage sequences within bacterial genomes and plasmids. The Comprehensive Antibiotic Resistance Database (CARD)^6^ (Jia et al., 2017) for identifying antibiotic resistance and virulence factors using the Resistance Gene Identifier (RGI) tool (under Perfect hit; Rigorous hit alone; and Perfect, Strict, and Loose hit criteria) was employed (Arndt et al., 2016). The ResFinder 4.1 server^7^ was used to predict the acquired antimicrobial resistance genes with a selected %ID threshold of 90.00% and the selected minimum length of 60% (Zankari et al., 2012) and/or chromosomal mutations (Bortolaia et al., 2020). Plasmid Finder 2.0 was used to search for mobile elements^8^ (Carattoli et al., 2014); virulence factor database (VFDB)^9^ was employed to predict putative virulence factors (Liu et al., 2019). PathogenFinder web server^10^ was used for the prediction of bacterial pathogenicity using as input the contig file in FASTA format (Cosentino et al., 2013).

### Functional Annotation

To conduct functional annotation, the InterProScan^11^ and the evolutionary genealogy of genes: Non-supervised Orthologous Groups (EggNOG) DB (Huerta-Cepas et al., 2019) tools were used. InterProScan v5.0 (Jones et al., 2014) classifies sequences into the family level and then analyses them on the basis of the databases that are registered with InterPro’s member databases signatures such as Pfam^12^, Conserved Domain Database (CDD)^13^, and TIGRFAM (a collection of manually curated protein families focusing primarily on prokaryotic sequences) (Selengut et al., 2007). After the process was concluded, EggNOG DB, provided by EMBL^14^, was employed for additional annotation (Huerta-Cepas et al., 2019). Additionally, the predicted genes were also annotated by several databases: Swiss-Prot^15^, MetaCyc (a database that contains pathways responsible for both primary and secondary metabolism)^16^ (Caspi et al., 2018), VFDB (a virulence factor database)^17^ (Chen et al., 2005), PHI: The Pathogen-Host Interaction database^18^, and Pfam^19^, through the Global Catalog of Microorganisms genome annotation project pipeline webserver^20^ (Wu and Ma, 2019).

### Kyoto Encyclopedia of Genes and Genomes Pathway Analysis

To identify the potential participation of the predicted genes of UTNGt21O strain in different biological pathways, genes were mapped to reference canonical pathways in the Kyoto Encyclopedia of Genes and Genomes (KEGG) (Macrogen Service Custom analysis, South Korea). The KEGG Orthology database of prokaryotes was used as the reference for pathway mapping (Nakaya et al., 2013).

### Pangenome Analysis

Pangenome analysis was carried out using the Roary tool version v1.007001 (Page et al., 2015) with MAFFT v7.427 aligner (Katoh and Standley, 2013) to cluster the genes encoding complete protein sequences into a core (hardcore and softcore) and accessory (shell and cloud) genomes. Genomic data of *W. cibaria* (9) and *Leuconostoc* (1) strains examined in this study were obtained from the NCBI online database (Supplementary Table 1). It was also used to search for the presence or absence of core-specific genes and sample genes.

### The Effect of Peptide Extract on the Cell Membrane Integrity of *Staphylococcus aureus* ATCC1026

The overnight bacterial suspensions of *S. aureus* ATCC1026 (1 × 10^5^ CFU/ml) grown in Brain Heart Infusion (BHI) broth (Merck Millipore, MA, United States) were washed twice with 1× phosphate-buffered saline (PBS) (pH 7.5) and treated with 1×, 1.5×, and 2× minimum inhibitory concentration (MIC) of UTNGt21O peptide extract and subsequently incubated for 24 h at 37°C. The production of peptide extract and MIC was described previously (Tenea et al., 2020b). In brief, the overnight *W. cibaria* UTNGt21O culture was used to extract cell-free supernatant (CFS) by centrifugation at 13,000 × *g* for 30 min (4°C). The CFS was filtered using a 0.22-μm porosity syringe filter (# STF020025H, ChemLab Group, United States), and 80% ammonium sulfate was added to precipitate the peptides that were recovered in 25 mM ammonium acetate (pH 6.5), desalted by using a Midi Dialysis Kit (cat # PURD10005-1KT, Sigma-Aldrich Co., LLC., Saint Louis, MO, United States) pre-equilibrated with phosphate buffer (pH 7.0) and stored at –20°C before use in further experiments. Titer, estimated as arbitrary units per milliliter, is defined as the highest dilution that inhibited the growth of the target strain and was determined by agar well diffusion method (Arena et al., 2018). The MIC was determined by microdilution broth method and was defined as the minimum peptide concentration that inhibits 50% of the target cells after counting the viable bacteria in plate count following the treatment with the peptide extracts at different concentrations (ranged from 800 to 12,800 AU/ml) compared with bacterial growth without any peptide added (Arena et al., 2018). The MIC (1×) was determined as 1,600 AU/ml. Bacterial cell culture without peptide treatment was used as the control. Furthermore, the DNA/RNA molecules were detected by electrophoresis in 1% agarose gel with ethidium bromide, running in 1× Tris–borate–ethylenediaminetetraacetic acid (EDTA) (TBE, pH 8.0) buffer (Sigma-Aldrich Co., LLC., Saint Louis, MO, United States) after extraction with chloroform (1:1, *v*/*v*), and precipitated with isopropanol and ammonium acetate (3 M).

### Whole Protein Profile of *Staphylococcus aureus* ATCC1026 Upon the Treatment With UTNGt21O Peptide Extract

The effect of UTNGt21O peptide extract on the whole target protein profile was analyzed using the SDS-PAGE method (Patra et al., 2015). Samples containing *S. aureus* in BHI (Merck Millipore, Burlington, MA, United States) broth were incubated with 1×, 1.5×, and 2× MIC of UTNGt21O peptide extract at 37°C for 24 h. Untreated cell with the peptide extract was used as a negative control. The cell pellet was suspended in 1× SDS-PAGE loading buffer, boiled for 5 min at 100°C, and centrifuged at 300 × rpm. The supernatant of treated and untreated cells with the peptide extract was used in SDS-PAGE. The tricine–SDS-PAGE method using RunBlue Bis-Tris Protein Gels (12%) and Dual Cool Mini-Vertical PAGE/Blotting Systems (Expedeon, Abcam, Cambridge, MA, United States) was used. The gel was stained with InstantBlue ready-to-use stain (Expedeon, Abcam, Cambridge, MA, United States) using a protocol recommended by the manufacturer.

## Results and Discussion

### Taxonomic Classification

After the complete genome or draft genome was assembled, a BLAST analysis was carried out to identify to which species each scaffold showed similarity (Altschul et al., 1990). The similarity proportions on the basis of the genus level as a result of the best hits for the entire contig were 54.84% with *Weissella*, 6.45% with *Leuconostoc*, 3.23% with *Lactococcus*, and 35.48% no match. Because the BLAST analysis depends on registered information, it is difficult to determine the information of the species that are not registered (Tenea et al., 2020b). In particular, the assembly results could be matched to a relative species or evolutionarily distant species due to sequence differences or errors that may occur during the assembly process. Therefore, more appropriate was to use the analysis results to identify patterns rather than to use it as an absolute criterion for species determination. The best top five hit results were identified (Supplementary Table 2). The five top genome results on the basis of ANI analysis are presented in Table 1. The closest genome was *W. cibaria* SP7 (GCF_004521965.1) with 86.21% ANI and 3.2% alignment coverage. Peculiarly, this strain was isolated from dairy cows of Kuwait (Patrone et al., 2020). At the time we first analyzed the UTNGt21O genome sequence similarity (Tenea et al., 2020b), the genome sequence and assembly of SP7 were not available. Hierarchical clustering of the data in two dimensions was represented by dendrograms, constructed by a simple linkage of ANI percentage identities (Supplementary Figure 1A). Although there is no standard accepted interpretation of species by alignment coverage, this analysis identifies a single clade corresponding exactly to UTNGt21O, with a minimum aligned genome length above 50% (Supplementary Figure 1B).

**TABLE 1:** The top five closest genomes resulting from ANI analysis.

Ranking	Similar genome: reference sequence assembly genome accession number NCBI	ANI (%)	Alignment coverage (%)
1	GCF_004521965.1_ *W. cibaria*_SP7	86.21	3.20
2	GCF_004521955.1_*W. cibaria*_SP19	85.98	3.44
3	GCF_015551795.1_*W. cibaria*_1001713B170221_170320	85.98	3.38
4	GCF_000878205.1_*W. cibaria*_MG1	85.95	3.39
5	GCF_014061105.1_*W. cibaria*_YRK005	85.92	3.54

*Ranking: the order of the highest ANI value; similar genome: species name that has high ANI value; ANI: percentage of relatedness at whole-genome level; alignment coverage: percentage of coverage by assembly sequence alignments against compared genome; ANI, average nucleotide identity.*

### Gene Prediction and Annotation

**FIGURE 1:**
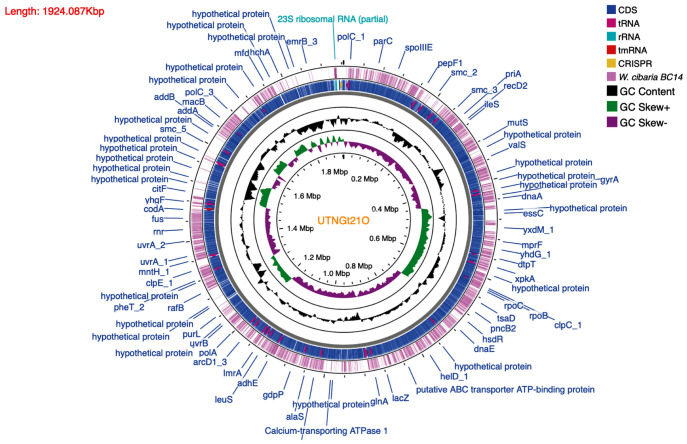
Genome map circular view of *W. cibaria* UTNGt21O generated using CGView Server (http://cgview.ca). The contents are arranged in feature rings (starting with outermost ring): The outermost first ring represent the map of reference taxa *W. cibaria* BC14; the second ring shows the CDS (coding sequences) with Prokka annotation (both strands combined); tRNA, rRNA, and tmRNA are indicated; the third ring displays the G+C content; the fourth ring shows the G/C skew information in the (+) strand (green color) and (–) strand (dark pink color); CRISPR are marked (yellow arrow).

After the complete genome was assembled, the locations of protein genes were predicted, and their functions were annotated. The genome contains 1,925 genes, 1,867 protein-coding genes (CDS), 78 tRNAs, eight rRNA (six copies of 5S rRNA, one copy of 16S rRNA, and one copy of 23S rRNA), and one copy of tmRNA. A physical genomic map of the UTNGt21O strain is shown in Figure 1. The gene prediction statistics are presented in Supplementary Table 3. Prokka was used to predict the location while BLAST was used to know about the function and identification of assembled sequences against nucleotide and protein sequence database. Predicted genes in the previous step were aligned with several databases to obtain their corresponding annotations with the aligners (Table 2). To ensure the biological meaning, the highest quality alignment result was chosen as gene annotation. On the basis of the annotation results, 107 proteins were predicted to be responsible for replication, recombination, and repair; 108 responsible for transcription; 103 responsible for carbohydrate transport and metabolism; 88 for the cell wall, membrane, and envelope biogenesis; 31 responsible for defense mechanisms; eight responsible for the biosynthesis of secondary metabolites, transport, and catabolism; and 438 has an unknown function. The gene-encoding proteins and their predicted functions are displayed in Table 3.

**TABLE 2:** Summary of the genome annotation performed by aligning gene sequences of the UTNGt21O strain to the sequences from several databases.

Sample	Number of genes (#)	CARD	MetaCyc	PHI	VFDB	Swiss-Prot	Pfam	KEGG	COG
Gt21O_contigs	1,867	33 (1.76%)	271 (14.45%)	94 (5.01%)	62 (3.31%)	705 (37.60%)	1,444 (77.01%)	1,409 (75.15%)	1,184 (63.15%)

*CARD, Comprehensive Antibiotic Resistance Database; MetaCyc, a database that contains pathways involved in both primary and secondary metabolism; PHI, The Pathogen-Host Interaction database is a biological database that contains curated information on genes experimentally proven to affect the outcome of pathogen–host interactions (http://www.phi-base.org/searchFacet.htm?queryTerm); VFDB, virulence factor database (http://www.mgc.ac.cn/VFs/main.htm); Swiss-Prot (https://www.uniprot.org/statistics/Swiss-Prot); pFam, a database with a large collection of protein families, each represented by multiple sequence alignments and hidden Markov models (HMMs) (http://xfam.org); KEGG, Kyoto Encyclopedia of Genes and Genomes; COG, Clusters of Orthologous Groups of proteins. Number of genes and percentage is shown.*

**TABLE 3:** EggNOG category distribution of functional annotation result.

EggNOG	Description	Count	Ratio (%)
J	Translation, ribosomal structure, and biogenesis	132	8.0734
A	RNA processing and modification	0	0.0000
K	Transcription	108	6.6055
L	Replication, recombination, and repair	107	6.5443
B	Chromatin structure and dynamics	0	0.0000
D	Cell cycle control, cell division, and chromosome partitioning	22	1.3456
Y	Nuclear structure	0	0.0000
V	Defense mechanisms	31	1.8960
T	Signal transduction mechanisms	39	2.3853
M	Cell wall/membrane/envelope biogenesis	88	5.3823
N	Cell motility	3	0.1835
Z	Cytoskeleton	0	0.0000
W	Extracellular structures	0	0.0000
U	Intracellular trafficking, secretion, and vesicular transport	24	1.4679
O	Post-translational modification, protein turnover, and chaperones	44	2.6911
C	Energy production and conversion	53	3.2416
G	Carbohydrate transport and metabolism	103	6.2997
E	Amino acid transport and metabolism	66	4.0367
F	Nucleotide transport and metabolism	72	4.4037
H	Coenzyme transport and metabolism	34	2.0795
I	Lipid transport and metabolism	44	2.6911
P	Inorganic ion transport and metabolism	72	4.4037
Q	Secondary metabolite biosynthesis, transport, and catabolism	8	0.4893
R	General function prediction only	147	8.9908
S	Function unknown	438	26.7890
Total	–	1,635	100

*Count: number of genes; ratio (%): percentage of genes.*

#### Probiotic Features Genes

Previously, we showed that LAB strains isolated from wild fruits possess a unique portfolio of enzymes that allow them to metabolize various compounds and present specific metabolic traits as a result of their microenvironmental origin (Tenea et al., 2020a). However, the presence of many gene sequences responsible for carbohydrate and amino acid transport and metabolism is considered an important probiotic characteristic. The annotation showed the presence of genes encoding for proteins participating in lactose and raffinose permease utilization, putative amino acid permease (protein YvbW), and inner membrane ABC transporter permease (protein YdcV). A putative allantoin permease belonging to a superfamily of AA permeases responsible for amino acid transport and metabolism was detected in the UTNGt21O genome. These proteins were found in several *W. cibaria* strains and related to probiotic potential (Falasconi et al., 2020). Besides, maltose phosphorylase (*malP*) was annotated. Maltose was found in environments where starch metabolic by-products are present such as a gut; thus, the presence of these enzymes can be putative indicators of a gut-adapted microorganism (Fontana et al., 2019). Genome analysis showed the presence of the gene *glpK_1*, encoded for glycerol kinase, a key enzyme participating in the regulation of glycerol uptake and metabolism. The ability of UTNGt21O strain to metabolize glycerol and to utilize the α-galacto-oligosaccharides (αGOS) family, d-melibiose [α-Gal-(1→6)-Glu], as well as the raffinose-family oligosaccharide (RFO) d-raffinose was previously detected by employing biochemical miniaturized fermentation tests (Tenea et al., 2020a). The results suggested the capacity of this strain to adapt to different niches because of its high genome flexibility, which points this strain as good candidates for functional food design or other biotechnological applications.

Genes encoding for phosphoenolpyruvate-dependent sugar phosphotransferase system (PTS), mannose-specific EIID component, PTS sorbose-specific EIIC component (*manZ_1*, *sorC_1, manX*), and PTS mannose-specific EIIAB component were also detected in the genome of UTNGt21O strain. Additionally, a single-gene copy *bglF* and *crr*, encoded for PTS beta-glucoside-specific EIIBCA component and PTS glucose-specific EIIA component, respectively, were detected. The PTS is a multiprotein system responsible for the regulation of a variety of metabolic and transcriptional processes (Meins et al., 1993).

Same as the genome of *W. cibaria* SP7 strain (Patrone et al., 2020), the UTNGt21O strain harbors genes encoded for enzymes predicted to participate in N-acetylglucosamine catabolism and synthesis (*nagA*, *nagB*, *nagC*, *glmS*, *glmM*, and *glmU*) as well as genes responsible for the rhamnose biosynthesis (*rflB* and *rmlD*). A hypothetical protein encoding for alcohol dehydrogenase was detected with 97.32% identity with a zinc-binding alcohol dehydrogenase family protein from *Weissella oryzae*. These genes were associated with the putative probiotic potential of *W. cibaria* SP7 strain (Patrone et al., 2020). Along with these sugar metabolism genes, other genes participating in stress resistance and cell adhesion such as *dnaK* and *brpA* were predicted in the genome of UTNGt21O that might contribute to the potential probiotic trait.

Gene prediction with Prokka identifies a single-copy gene *brpA* and *tagU* encoded for a biofilm regulatory protein A and polyisoprenyl-teichoic acid–peptidoglycan teichoic acid transferase TagU. These genes showed 97.24 and 99%, respectively, sequence identity with LCP protein family from *W. oryzae*; LCP is a transcriptional regulator (COG 1316) responsible for cell wall, membrane, and envelope biogenesis (Kawai et al., 2011). Also, a single-copy gene, *icaA*, encoded for poly-beta-1,6-N-acetyl-D-glucosamine synthase was detected in the UTNGt21O genome. This gene was previously detected in *W. confusa*, encoded for a surface polysaccharide whose presence was associated with biofilm formation (Little et al., 2014).

#### Defense Mechanism Genes

Gene prediction highlighted the presence of a specific gene *hslO* encoded for the 33-kDa chaperonin, a redox-regulated molecular chaperone, which plays an important role in defense toward oxidative stress. This protein belongs to the Hsp33 family of molecular chaperones, showing 64.41% sequence identity with a heat shock protein Hsp33 of several *Weissella* species. The UTNGt21O strain harbored a putative cold shock protein CspB, which might explain the adaptation to different environments. A hypothetical protein, phage shock protein C (COG1983), involved in cellular processes of adaptation to atypical conditions, was also detected. The members of this protein family contain a PspC conservative domain, as do other members of the larger family of proteins described by pfam04024.

A hypothetical protein, RelB antitoxin, a component of a type II toxin–antitoxin (TA) system, with a role in the defense mechanism, was also found to have 92.86% identity with a type II toxin RelB/DinJ system of *Weissella muntiaci*. Four specific genes (*pbpB*, *pbpF*, *ponA*, and *penA*) encoding for penicillin-binding proteins and two genes (*rnj1* and *rnj2*) responsible for the resistance to toxic compounds and beta-lactam antibiotics were found in the genome of UTNGt21O strain.

A putative pesticidal crystal protein Cry22Aa was found in the UTNGt21O genome only but not in other *W. cibaria* strains. BLASTP protein sequence homology search and protein alignment showed a 92.16% identity with a hypothetical protein from *W. oryzae*. This protein contains a domain DUF5011 with an unknown function. Previous studies showed that this protein from *Bacillus thuringiensis* subsp. *kurstaki* has a toxic effect on several insect larvae (Elgizawy and Ashry, 2019).

A hypothetical protein belonging to the superfamily of HNH (His-Asn-His) endonucleases, with a function in the defense mechanism, was annotated with Prokka. These proteins are small nucleic acid-binding proteins that are generally associated with endonuclease activity. BLASTN protein sequence search indicated a 52.27% identity with an HNH endonuclease from *W. cibaria* (accession no. WP_060654567.1). A hypothetical protein, hydrolase family 25, belonging to a superfamily of the endo-N-acetylmuramidases (muramidases) enzymes, was detected in the UTNGt21O genome. The protein sequence has a 67% identity with a terminase large subunit from *Lactococcus lactis* (accession no. NYZ60031.1) and a 48.89% identity with an endolysin from *Weissella koreensis* (accession no. WP_104914412.1). Endolysins generally have narrow substrate specificities with either intraspecies or intragenus bacteriolytic activity having putative conservative domains: GH25 muramidase superfamily and terminase_1 superfamily. Besides, a gene (*acm*) encoding for lysozyme M1 and *lyc* encoding for autolytic lysozyme (COG3757) were detected. These proteins are located in the cell wall, which degrades bacterial cell walls by catalyzing the hydrolysis of 1,4-beta-linkage between N-acetylmuramic acid and N-acetyl-D-glucosamine residues, found in *Lactobacillus fermentum* and *L. lactis* (Rau et al., 2001). Within the *Weissella* taxa, the function of these proteins remains unknown.

Gene prediction displayed four genes encoding for ABC transporter ATP-binding protein and several putative ABC transporter permeases, but not genes for bacteriocin biosynthesis. The ABC transporter permeases were classified as membrane proteins harboring specific domains such as DUF1275 of an uncharacterized membrane protein YoaK (COG3619) and DUF161 and DUF2179 domains of an uncharacterized membrane-anchored protein YitT (COG1284).

Nonetheless, in a more in-depth characterization with BAGEL4, a related gene cluster for bacteriocin production was detected. A specific bacteriocin, Enterolysin_A, was found in the UTNGt21O genome (Tenea et al., 2020b). No Enterolysin_A-encoded gene was predicted in other *W. cibaria* strains. We showed that this putative bacteriocin is larger (17 kDa) in size and when combined with EDTA inhibits the growth of the two indicator strains, *E. coli* and *Salmonella enterica*, at both the early and later logarithmic phase of growth (Tenea et al., 2020b). Previous research indicated that *W. cibaria* 110 produce a class IId bacteriocin, weissellicin 110 (Wu et al., 2015), while the strains SP7 and SP19 harbored colicin V bacteriocin (Patrone et al., 2020). The genome information presented here will help further specific studies of this strain and exploit its probiotic and antimicrobial potential.

### Prediction of CRISPR, Prophage, Antibiotic Resistance Genes, Virulence Factors, and Pathogenicity

The results obtained from the CRISPR finder database indicated that the UTNGt21O genome contains one potential CRISPR arrays found within contig 5 (beginning at 121,014–ending at 121,114) and one putative CAS sequence (Cas3_0_1) within contig 8 (beginning at 77,077–ending at 78,147) (Supplementary Table 4). CRISPR array comprised a short spacer sequence incorporated between degenerate repeats (DR consensus) with 96% conservation repeats and 100% spacer conservation. The DR sequence was found in several *Leuconostoc* spp. and *Lactobacillus* spp. strains according to BLAST analysis. Cas3 is a single-stranded DNA nuclease that functions as an ATP-dependent helicase in the CRISPR-CAS immune system (Sinkunas et al., 2011). The closest genome *W. cibaria* SP7 does not show any CRISPR-Cas genes, while *W. cibaria* SP19 strain harbored a type 2 CasA protein (Patrone et al., 2020).

The presence of mobile genetic elements such as prophages, integrases, and insertion sequences (ISs) in bacteria is considered the primary contributing factor to genetic diversity and niche adaptation (Fontana et al., 2019). PHASTER analysis revealed a total of five prophages within the UTNGt21O genome; one prophage intact region and one incomplete within contig 1, and three prophage regions have been identified in contig 10, of which one region was intact and two regions were incomplete (Table 4). The length of the intact prophage varies from 18.5 to 22.9 kb. The presence of intact regions might help against the invasion of other prophages (Falasconi et al., 2020). No mobile elements were found with PlasmidFinder (selected %ID threshold: 95.00%, selected minimum length: 60%), which indicates the stability of the UTNGt21O genome.

**TABLE 4:** Prophage regions identified within contigs 1 and 10 of UTNGt21O genome using the PHASTER webserver.

Contig	Region	Region length	Completeness	Score	# of total proteins	Region position	Most common phage	GC%
Contig 1	1	22.9 kb	Intact	140	27	185771–208685	PHAGE_Lactococ_lato_NC_004746 (6)	37.02
Contig 1	2	33.6 kb	Incomplete	30	36	210943–244602	PHAGE_Lactob_J_1 NC_022756 (3)	38.32
Contig 10	1	18.3 kb	Incomplete	30	27	4994–23322	PHAGE_Lactob_phiadh_ NC_000896 (3)	35.22
Contig 10	2	18.5 kb	Intact	110	19	26408–44909	PHAGE_Lactob_JCL1032_ NC_019456 (3)	36.77
Contig 10	3	7 kb	Incomplete	10	22	52202–59234	PHAGE_Clostr_phiCD119_ NC_007917 (3)	36.42

*Region: the number assigned to the region; region length: the length of the sequence in that region; completeness: a prediction of whether the region contains an intact or incomplete prophage based on the criteria: intact (score > 90%); questionable (score 70–90); incomplete (score < 70%); score: the score of the region on the basis of the mentioned criteria; # of total proteins: the number of ORFs present in the region; region position: the start and end positions of the region on the bacterial chromosome; most common phage: the phages(s) with the highest number of proteins most similar to those in the region; GC %: the percentage of GC nucleotides of the region.*

From the safety perspective, an analysis of the antibiotic resistance pattern is important when characterizing a new isolate designed for probiotic use. In this study, UTNGt21O strain genes were annotated with CARD protein ID and grouped by drug class, resistance mechanism, and AMR gene family. The number of genes in each category is shown in Supplementary Table 5. The genome of *W. cibaria* UTNGt21O was found to contain several putative resistance genes associated with resistance to triclosan (1), streptomycin (1), para-aminosalicylic acid (1), nybomycin (1), mupirocin (1), monobactam (1), macrolide (1), elfamycin (1), cephamycin (1), carbapenem (1), rifamycin (2), pleuromutilin (2), phenicol (2), penam (2), glycopeptide (2), fusidic acid (2), aminocoumarin (2), acridine dye (2), aminoglycoside (3), lincosamide (3), tetracycline (5), and peptide (7). As reported by Munoz-Atienza et al. (2013), 60% of *Weissella* species are antibiotic-resistant. Nonetheless, the antibiotic resistance studies in *Weissella* are limited due to the high divergence among the resistance genes. In this study, a gene, *macB*, encoding for macrolide export ATP-binding/permease protein MacB, involved in the efflux of macrolide (COG0577), was annotated with the EggNOG database. The protein sequence had 97.85% identity with the ABC transporter ATP-binding protein/permease from *W. oryzae* (WP_027699581.1) and 70.20% identity with the ATP-binding cassette domain-containing protein from *W. cibaria* (WP_195555901.1). After Munoz-Atienza et al. (2013), in the genome of *W. cibaria* of aquatic origin, *mef*(A/E) genes are participating in the active efflux of macrolides.

In other studies, the genome analysis of *W. cibaria* 110 indicated the presence of several antibiotic-resistant genes that encode putative vancomycin and tetracycline, suggesting that it is a multidrug-resistant strain (Li et al., 2017). The inborn vancomycin resistance of the *Weissella* taxa and other LAB such as *Lactobacillus*, *Pediococcus*, and *Leuconostoc* species was already disclosed and refer to the absence of D-Ala-D-lactate in their cell wall, which is the target of vancomycin A (Ammor et al., 2007; Kamboj et al., 2015). Nonetheless, such resistance cannot be attributed to the acquisition of resistance genes. In our study, the gene *vanRA* with 44.5% sequence identity with *Enterococcus faecium* was predicted with the CARD database, but its level of expression is not known. Based on genome analysis, UTNGt21O is a multidrug-resistant strain. Nonetheless, in addition to the *in silico* genome analysis, the complementary *in vitro* analysis of the antibiotic susceptibility of some common antibiotics performed following the FEEDAP document (EFSA, 2012) indicated that the UTNGt21O strain was susceptible to ampicillin, amoxicillin, cefotaxime, tetracycline, erythromycin, and penicillin and resistant to gentamycin (Supplementary Table 6). In this study, the resistance to gentamycin was not predicted with the CARD database. Previous studies reported resistance to gentamicin, kanamycin, and norfloxacin in food-associated weissellas (Patel et al., 2012). More recently, a safety study of *W. cibaria* SP7 and SP19 strains indicated that these species were susceptible to several antibiotics (Patrone et al., 2020). On the basis of the EggNOG genome annotation, a gene (*femA*) encoding for methicillin resistance protein was detected in the genome of the UTNGt21O strain. The presence of the methicillin gene along with fosfomycin and sulfonamide-resistant genes within the *Weissella* genus was previously described (Abriouel et al., 2015). Methicillin resistance protein is often found in *Staphylococcus* species; its presence on the *Weissella* genome may suggest a horizontal gene transfer. Nonetheless, its level of expression is not known yet. In contrast with the study of Abriouel et al. (2015), no fosfomycin and sulfonamide-resistance genes were detected in the UTNGt21O genome. The ResFinder analysis (selected %ID threshold: 98.00%, selected minimum length: 60%) did not detect any acquired antibiotic resistance genes, indicating the strain genome stability.

Some putative virulence factor genes were predicted with the virulence factor database (Supplementary Table 7). Out of the 62 genes predicted, nine were not annotated with VFDB. However, neither genes related to collagen adhesin proteins nor encoded for a mucus-binding protein were detected in the UTNGt21O genome. Previous studies indicated the presence of genes encoding for collagen adhesin proteins in the genome of some *Weissella cebi* strains and *W. confusa* strain LBAE C39-2 (Abriouel et al., 2015). Any genes associated with the toxin production system including botulinum neurotoxin homolog from *W. oryzae* SG25 (Panthee et al., 2019) were detected in the UTNGt21O genome. In the present study, some genes encoded for putative enolase (*eno*), pyruvate dehydrogenase E1 component, beta subunit (*pdhB*), response regulator factor (*mprA*), etc. were predicted in the category of virulence factors. These genes are involved in biological processes such as adaptation to environmental changes (*mprA*), enolase is essential for the degradation of carbohydrates via glycolysis (carbohydrate metabolism according to KEGG pathway analysis), and the dehydrogenase complexes responsible for energy production and conversion. However, their function on virulence is not known. Two genes, *hlyD* and *hly*III, were predicted as “probable hemolysin” with 51.6% identity, and a channel protein of the hemolysin III family with 41.6% identity, but further studies need to confirm whether these genes are expressed or not. In line with previous findings (Patrone et al., 2020), correlative *in vitro* analysis evaluating the cell growth on blood agar plates indicated neither β-hemolytic nor α-hemolytic phenotypes (data not shown). From the safety point of view, the UTNGt21O strain does not harbor adhesins or aggregation substances; at this point, we cannot appreciate that the detected putative virulence factors may induce human infections. Besides, no genes associated with human pathogens were predicted with the PathogenFinder. The probability of being a pathogenic strain was low (<0.048 out of 1); however, this strain was predicted as a non-human pathogen, thus a “secure strain.”

### EggNOG Functional Annotation Disclosed Novel Genes With Biotechnological Significance

**FIGURE 2:**
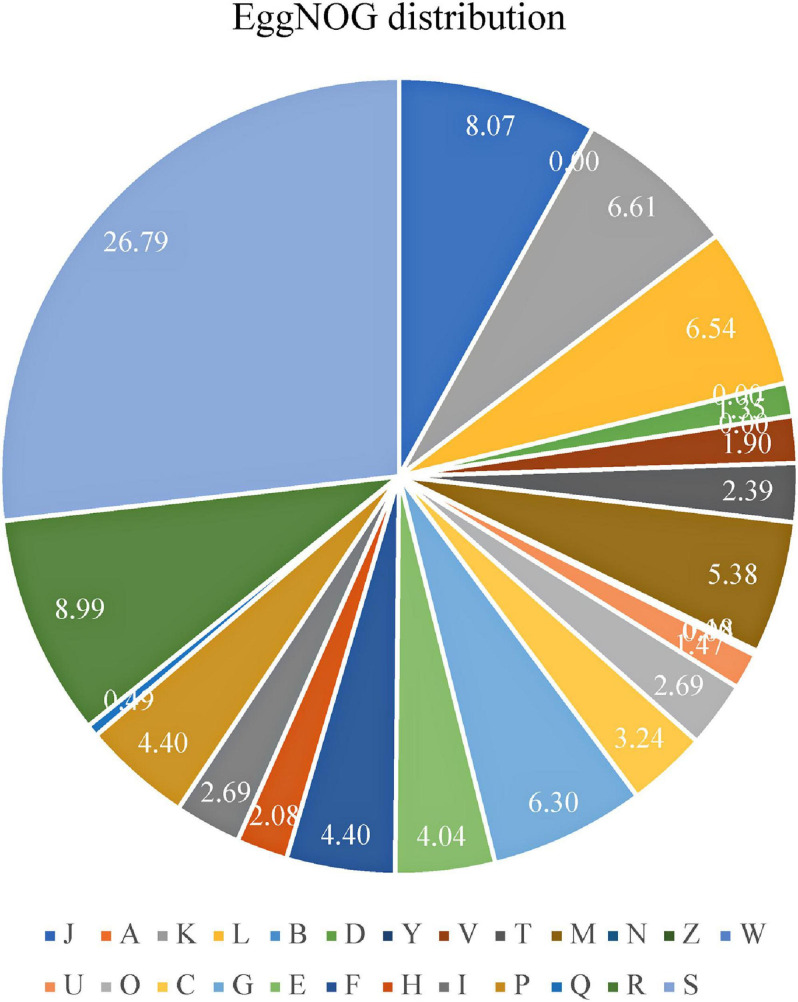
Proportion of EggNOG genes distributed according with their functional annotation as described in Table 3.

Annotation was refined with EggNOG on the basis of the input of the protein sequences predicted with Prokka. Among 1,867 total proteins, 1,620 matched EggNOG DB proteins (1,605 single EggNOG and 15 multi EggNOG proteins) and 247 proteins with no hit. However, 247 new proteins were found in the genome of the UTNGt21O strain. Figure 2 shows the EggNOG category distribution of functional annotation results and their frequency according to the description of Table 3. More than 26% of the proteins have an unknown function (438 proteins), while 8% (132 proteins) participate in the translation, ribosomal structure, and biogenesis.

Gene annotation with EggNOG indicated the presence of several enzymes from the family of isomerases, such as lysine racemase, maltose epimerase, and isoleucine 2-epimerase. However, 6.29% of proteins are responsible for carbohydrate transport and metabolism. A gene encoding for 3-beta-hydroxycholanate 3-dehydrogenase [NAD (+)] 1 was also detected in the genome of the UTNGt21O strain. BLASTN (non-redundant protein database) indicated 58.44 and 44.30% identity with an SDR family NAD(P)-dependent oxidoreductase from *Fructobacillus durionis* (WP_091501657.1) and *W. cibaria* (WP_134658903.1), respectively. This enzyme was found to be responsible for the modification of secondary bile acids into iso-bile acids (3 beta-bile acids) via epimerization of the 3-OH group through a 3-oxo intermediate (Devlin and Fischbach, 2015). The protein displays a conserved FabG domain and belongs to a superfamily of anticapsin BacC. Members of this family are dihydroanticapsin 7-dehydrogenase (EC 1.1.1.385), one of the seven key molecular markers for the biosynthesis of the non-cognate amino acid anticapsin, a building block for the dipeptide antibiotic natural product bacilysin (Ozcengiz and Ogulur, 2015).

A putative gene encoding for penicillin acylase (EC 3.5.1.11) was detected in the UTNGt21O genome. BLASTN (PDB protein database, which contains information about experimentally determined structures of proteins, nucleic acids, and complex assemblies) analysis indicated a 30% amino acid identity with penicillin V amidase from *Lysinibacillus sphaericus* (Supplementary Figure 2). No homologous was found in other *W. cibaria* strains. The pairwise analysis of the query (penicillin acylase from UTNGt21O) and the database sequence search detected 17 hits in the database. The tree view based on pairwise alignment is shown in Supplementary Figure 3. A conserved domain hits Ntn hydrolases, a superfamily of enzymes that are activated autocatalytically via an N-terminally located nucleophilic amino acid. This family of hydrolases includes penicillin acylase, the 20S proteasome, alpha and beta subunits, and glutamate synthase that catalyze the hydrolysis of amide bonds in either proteins or small molecules, and each one of them is synthesized as a pre-protein. Penicillin acylase is one of the most studied enzymes because of its industrial significance, being also a model for the study of prokaryotic expression control and post-translational processing (Illanes and Valencia, 2017). Penicillin V acylase, also known as conjugated bile salt acid hydrolase (CBAH), catalyzes the hydrolysis of penicillin V to yield 6-amino penicillanic acid (6-APA), an important key intermediate of semisynthetic penicillins (Rossocha et al., 2005).

A gene encoding for the biosynthesis of riboflavin protein RibD (*ribD*) was detected in the genome of UTNGt21O. BLASTN protein analysis indicated a specific hit (*E* value: 1.4e-58) to deoxycytidylate deaminase (COG2131), having a catalytic motif and zinc-binding sites. This protein showed 77.99% sequence identity with a competence ComE protein 2 of *W. cibaria* (WP_099087665.1).

A hypothetical protein, DSBA oxidoreductase, with 90.80% identity with *W. oryzae* and no putative conserved domain sequence was annotated in the genome of UTNGt21O. This protein was neither found in *W. cibaria* strains nor *Leuconostoc*. Also, two genes, *arsM* and *ubiE*, encoding for arsenite methyltransferase and ubiquinone/menaquinone biosynthesis C-methyltransferase UbiE were also detected. The proteins ArsA and UbiE showed 70.95 and 96.77% sequence identity with a putative protein from class I SAM-dependent methyltransferase of *W. koreensis* and *W. oryzae*, respectively, which catalyzes the methylation of one or more specific substrates (small molecules, lipids, nucleic acids, etc.) using S-adenosyl-L-methionine (SAM or AdoMet) as the methyl donor (Tran et al., 1998).

### Pathway Analysis

**FIGURE 3:**
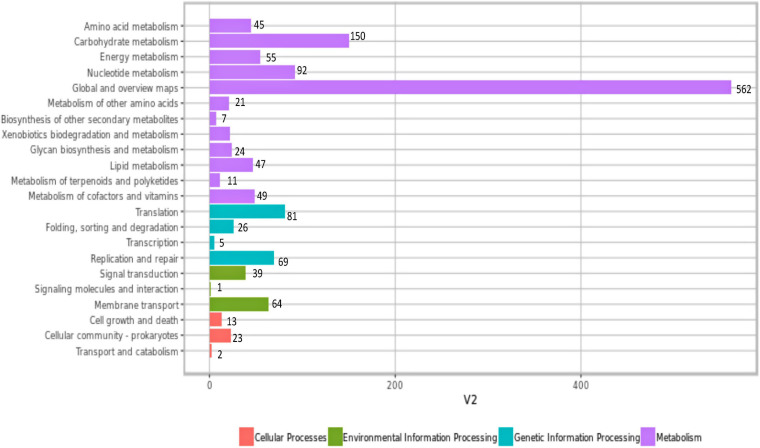
Mapping of the KEGG proteins according with their function. Genes are annotated with the KEGG protein ID first, and then, genes with the KEGG protein ID were mapped to the KO entry and further mapped to KEGG pathway. The number of genes in each category is shown. KEGG, Kyoto Encyclopedia of Genes and Genomes; KO, KEGG orthology.

Genes were annotated with the KEGG protein ID first, and then genes with the KEGG protein ID were mapped to the KEGG orthology (KO) entry and further mapped to the KEGG pathway. KEGG pathway analysis revealed that a total of 1,679 genes were found participating in 22 KEGG pathways in *W. cibaria* UTNGt21O (Figure 3). KEGG suggested that the highest number of genes encoding proteins are responsible for carbohydrate metabolism (150), nucleotide metabolism (92), energy metabolism (55), metabolism of cofactors of vitamins (49), lipid metabolism (47), biosynthesis of secondary metabolites (7), etc. Among them, a mannose-specific PTS component IID, PTS mannose transporter subunit IIAB, and PTS mannose transporter subunit IIC are important for probiotic potential. A triosephosphate isomerase (*tpiA*) was found responsible for carbohydrate metabolism, fructose and mannose metabolism, and inositol phosphate metabolism. Among the genes mapped within the group of metabolism of cofactors and vitamins/folate biosynthesis, two genes of the UTNGt21O (ID: 00314 and ID: 00831), annotated with KEGG gene ID: lack: FLP1508985, bifunctional folylpolyglutamate synthase/dihydrofolate synthase and gene ID: wcb: AO080_09625, respectively, were found encoding for the tetrahydrofolate synthase. The production of vitamins such as folate (vitamin B) by some lactobacilli is considered beneficial for the hosts in case of vitamin deficiency (Santos et al., 2008). Besides, the query ID: 00315, gene *ylgG* encoded for a hypothetical protein, was annotated with KEGG ID: lla: L168057, participating in the folate biosynthesis pathway.

Among the genes (562) grouped as global and overview maps in the KEGG pathway, a UTNGt21O query ID gene, *lmrA*, annotated with KEGG gene ID: lgv: LCGL_0296, a multidrug ABC transporter ATP-binding subunit, was mapped within the human diseases/drug resistance: antimicrobial, beta-lactam resistance, environmental information processing, and membrane transport/ABC transporters of KEGG pathway. This protein is a component of the defense mechanism and has a specific hit on MdlB superfamily proteins (COG1132). A gene *htrA* encoded for a serine Do-like protease HtrA, with KEGG ID: wcb: AO080_00675 serine protease, was mapped within the human disease, drug resistance, antimicrobial, cationic antimicrobial peptide (CAMP) resistance, environmental information processing, signal transduction, and two-component system. Serine proteases are periplasmic proteins of the S1-C subfamily (COG0265) having a C-terminal PDZ domain and function in post-translational modification, protein turnover, and chaperones. A hypothetical protein, UTNGt21O Query ID: 00795, was classified with KEGG ID: wcb: AO080_09105 exopolysaccharide biosynthesis protein, but no pathway was associated with this protein. Complementary *in vitro* analysis indicated no production of exopolysaccharide from glucose (40%) or sucrose (40%) by the UTNGt21O strain (data not shown).

### Pangenome Analysis

**FIGURE 4:**
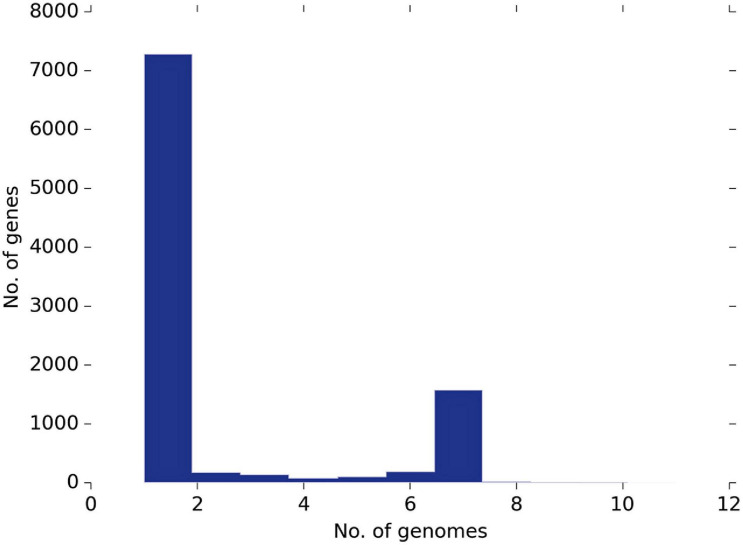
The pangenome frequency plot. The frequency of genes versus the number of genomes is described in Supplementary Table 1.

**FIGURE 5:**
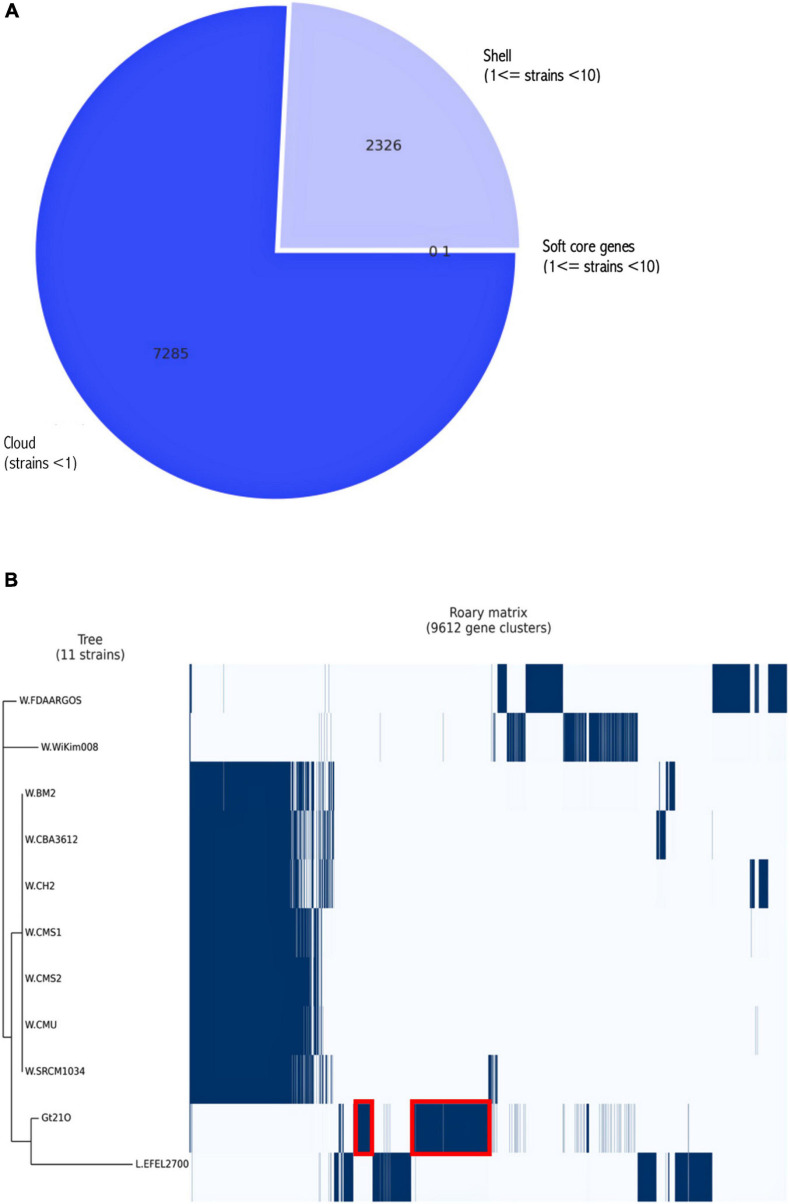
**(A)** The number of genes belonging to the core, the soft core, the shell, or the cloud of the UTNGt21O species is pictured as a pie chart. **(B)** Gene content comparison of the 11 considered strains. The matrix shows genes typical of each strain and those conserved in all. Two distinct clusters of the target strain are highlighted in red.

Based on the Roary pangenome pipeline analysis, 9,612 genes were analyzed concerning their distribution within the 11 strains. Figure 4 shows the frequency of genes versus the number of genomes. Among them, 7,285 genes were sample-specific genes (cloud genes), 2,326 (shell genes), and only one gene was common in 11 genomes (core genes), indicating the high genetic diversity (Figure 5A). These gene clusters were inspected to understand which peculiarities they bestow to each strain; the most interesting are highlighted in Figure 5B. However, we identified several species-specific gene-encoding proteins. By examining the EggNOG analysis, these proteins were grouped in the “S category” of unknown function and represent 26.78% of the genome.

The 50S ribosomal protein L34-encoding gene was found in all 11 genomes, while 50S ribosomal protein L30, methionine adenosyltransferase, and glutamine hydrolyzing GMP synthase genes were found in most of *W. cibaria* genomes but not UTNGt21O. The gene encoding for the transposase was found in *Leuconostoc* only.

The EggNOG analysis indicated that 31 gene-encoding proteins were found participating in the defense mechanisms. By comparing the presence/absence genes between the UTNGt21O strain and the 10 selected strains, several defense enzymes were found as strain specific. However, UTNGt21O harbors *hsdR* and *hsdM* genes encoding for enzymes responsible for defense mechanisms. The gene *hsdR* encoded for a putative type-1 restriction enzyme R protein with 85% sequence identity with DEAD-like helicase from *E. coli*, and 77.61% identity with a type I restriction endonuclease subunit R from *Streptococcus macedonicus*. The presence of these genes may suggest a horizontal gene transfer between the genera habituating the same niches. The gene *hsdM* encoded a type I restriction enzyme EcoKI M with 80.33% identity to type I restriction–modification system subunit M from *S. macedonicus*. According to the Pfam database (pfam02384), these enzyme hits a superfamily of N6-DNA methylases, a restriction–modification (R–M) system, that protect the bacterial cell against the invasion of foreign DNA by its endonucleolytic cleavage. Also, a hypothetical protein, CAAX self-immunity protein, was detected. This protein was found in the genome of *W. cibaria* 110, being responsible for self-defense against its bacteriocins (Li et al., 2017).

A multidrug ABC transporter ATP-binding protein and permease protein were detected in the genome of UTNGt21O as a component of the defense mechanism (Fath and Kolter, 1993). On the basis of BLASTN analysis, the gene *lmrA* encoding for multidrug ABC transporter was found in *L. lactis* subsp. *lactis* with 73.10% sequence identity and *W. confusa* strain N17 with 74.48% sequence identity. The pangenome analysis showed no presence of the *lmrA* gene in the 10 strains used in the comparison. A gene *lytA* encoding for autolysin, a peptidoglycan recognition protein (PGRP), a protein superfamily that hydrolyzes peptidoglycans of bacterial cell walls. These proteins harbor a conserved ZoocinA_TRD domain (pfam16775). There was no conserved ZoocinA_TRD domain homologous in other sequenced *W. cibaria* strains.

Two putative DNA repair helicases RadD (*radD*-1 and *radD*-2), encoding for type III restriction res protein subunit, and two genes (*dnpA* and *yhdJ*), belonging to type III restriction–modification system DNA endonuclease (EC 3.1.21.5), were also annotated in the genome of UTNGt21O strain but not the other genomes of *W. cibaria* or *Leuconostoc* strains used in the present study.

Taken together, the pangenome analysis suggested a great genomic variation among the compared species, which may correlate with a significant gene gain/loss during adaptation in different natural niches.

### The Peptide Extract of the UTNGt21O Strain Induced the Release of Aromatic Molecules DNA/RNA From the Target *S. aureus* ATCC1026 Strain

**FIGURE 6:**
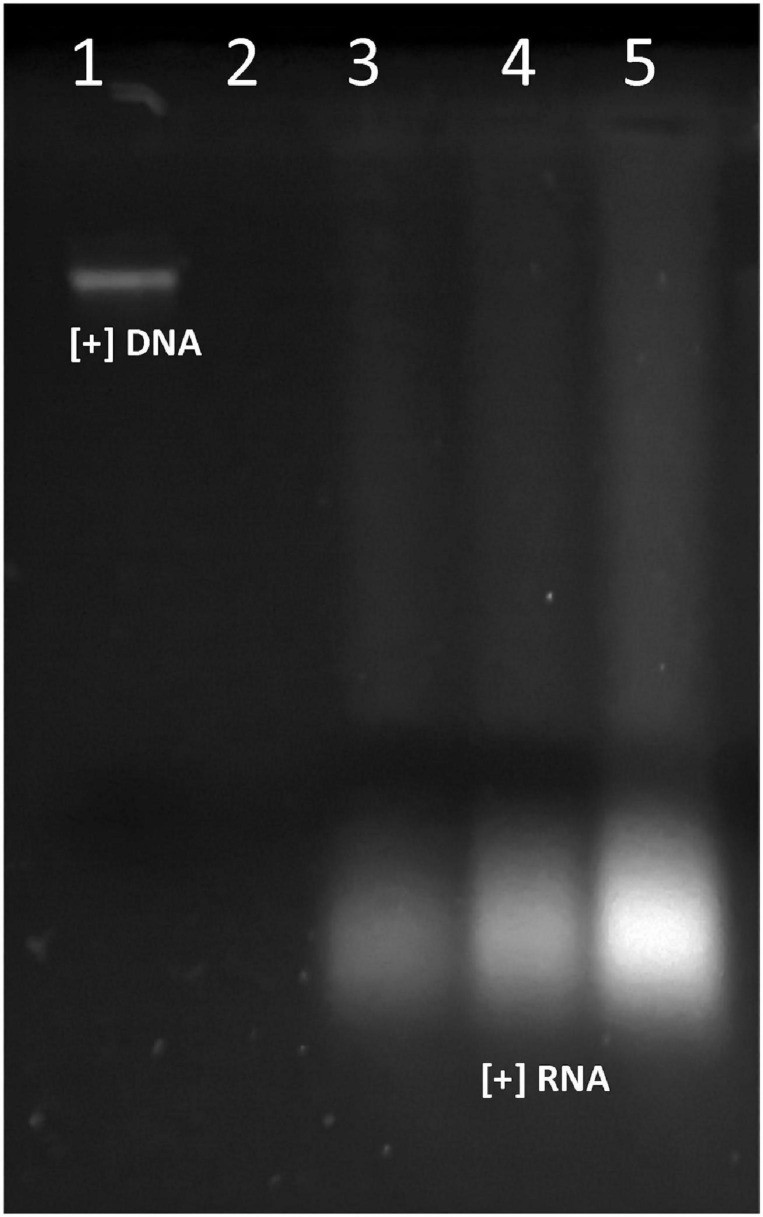
The effect of UTNGt21O peptide extract on *S. aureus* ATCC1026 cell membrane integrity. Legend: lane 1: *S. aureus* ATCC1026 genomic DNA (+); lane 2: *S. aureus* with no peptide treatment (absence of DNA/RNA molecules); lanes 3–5: RNA molecules detected when *S. aureus* was treated with 1×, 1.5×, and 2× MIC peptide extract.

Generally, *S. aureus* is found on human skin, and some species were detected as foodborne pathogens in fruits and fruit-derivative products (Strawn et al., 2011). Unlike Gram-negative bacteria, *S. aureus* produces toxins in food before being consumed and thus is an important food poison that cause human illness (Miao et al., 2016). Thus, it is our interest to identify natural antimicrobials that, when covering the fruits, might protect for contamination by microorganisms preserving their properties. The antimicrobial effectiveness against certain indicator bacteria depends on the producer strain, dose applied, and the mechanism of action; therefore, these characteristics should be demonstrated independently for each target microorganism designed to be used. In our previous research, the peptide extract from the UTNGt21O strain was found to induce cell damage and leakage of cytoplasmic molecules of *E. coli* ATCC25922 and *S. enterica* subsp. *enterica* ATCC51741, as the primary killing mechanism. The cell membrane alteration was dose dependent (Tenea et al., 2020b). In this study, we address the question if the peptide extract from UTNGt21O might compromise the cell membrane of a Gram-positive bacteria in the same manner as seen with Gram-negative bacteria. The incubation of *S. aureus* cells with different doses (1×, 1.5×, and 2× MIC) of the peptide extract resulted in the release of RNA molecules only (Figure 6), suggesting that the *Staphylococcus* membrane was susceptible to peptides, and its alteration and cell death occur at 24-h exposure. The absence of DNA might arise if the peptides bind the DNA molecules inducing double-stranded breaks and subsequent degradation, while the free RNA molecules might be protected by some other molecular mechanisms, but further investigations are needed to prove this statement. No DNA/RNA was detected in untreated control samples. This result was in agreement with our previous findings that some peptides disrupt the cell membrane of the target Gram-negative bacteria and their effectiveness was target specific (Tenea and Guana, 2019). Recent work on the synthetic peptide melamine mode of action against *S. aureus* indicates that the peptide interacted with the target cell membrane inducing membrane depolarization and leakage of ATP and DNA/RNA molecules followed by cell death, whereas another protein Mel4 does not induce cell membrane disruption inducing cell killing by autolysis mechanism (Yasir et al., 2019). However, the production of antimicrobial metabolites is considered an important feature by which probiotic species can reduce or diminish the pathogen growth in several niches. Thus, these metabolites may contribute to the overall increase of the food security and reduce the economic loss as a result of rapid contaminations of foods, strategies that are aligned with national and global requirements to reduce diseases associated with the presence of non-desirable microorganisms in different food matrices.

### Whole Protein Profile of *S. aureus* ATCC1026 Treated With Peptide Extract

**FIGURE 7:**
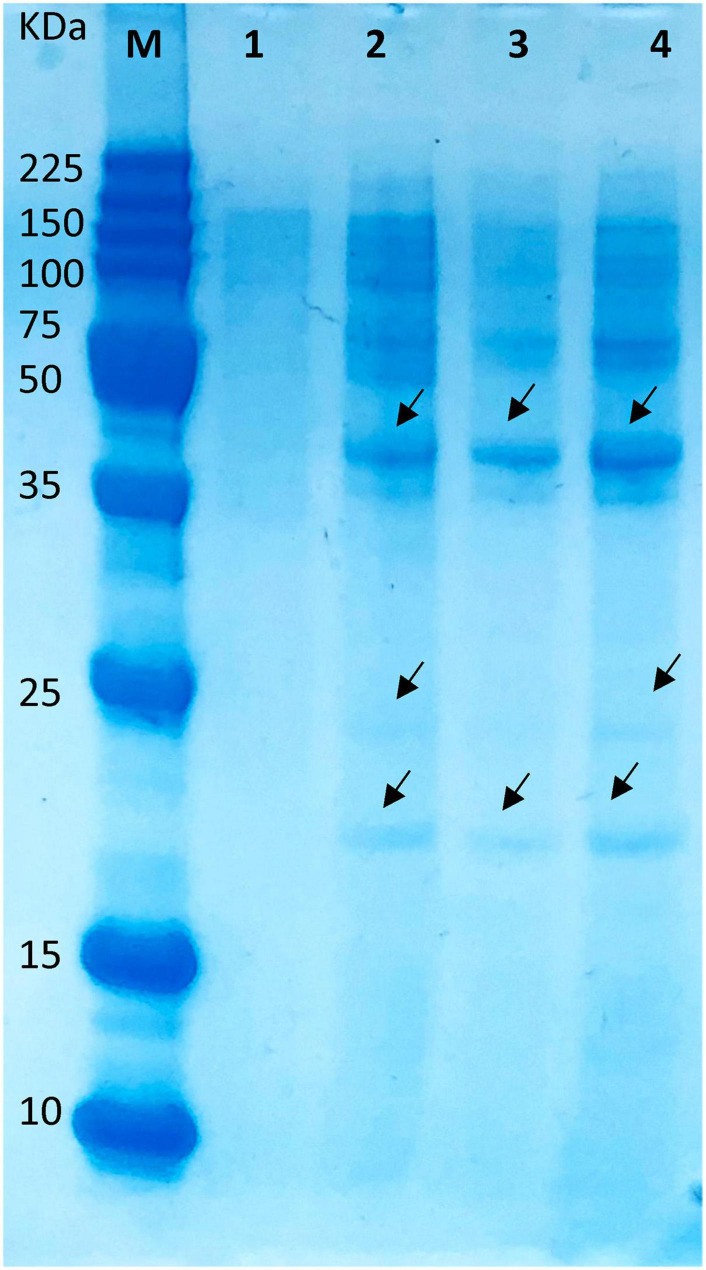
Different expression profiles of *S. aureus* ATCC1026 treated with peptide extract of UTNGt21O. Legend: lane 1, untreated *S. aureus* (control); lanes 2–4: 1×, 1.5×, and 2× MIC of UTNGt21O peptide extract at 24 h of incubation. M, molecular marker (broad range protein molecular weight marker, Promega #V8491). Arrows indicate different bands.

The interaction between the peptide and proteins from the target bacteria might result in a modification of the overall protein profile with the blockage of expression of lower and higher molecular weight proteins (Xue et al., 2016). As presented in Figure 7 (lane 1), a lower expression of high molecular weight proteins was detected in the *Staphylococcus*-untreated peptide, while the treatment with the different concentrations of the peptide disclosed the presence of both high and low molecular weight proteins (Figure 7, lanes 2–4). According to the visual estimation of the protein profile, a divergent electrophoretic pattern was detected. No difference in protein pattern was detected when different peptide concentrations were applied. These results were in concordance with our previous findings that some peptides might induce visible phenotypic changes in the whole protein of a target cell and might induce breaks to some extent in the membrane proteins (Tenea, 2020c), thus defining the possible bactericidal mode of action. These promising results will help in further developing antimicrobial formulations based on peptide extracts that might kill simultaneously both Gram-negative and Gram-positive microorganisms coexisting in the same food matrix, thus enhancing their safety.

## Conclusion

The whole-genome sequencing and genetic characterization of the *W. cibaria* UTNGt21O strain reveal its genetic diversity and the presence of novel genes encoding proteins with promising biotechnological properties. To the best of our knowledge, this is the first genome characterization of a lactic acid bacterium strain from the *Weissella* genus originating from wild *S. quitoense* Lam. (naranjilla) shrub. Based on the genome analysis, the results indicated that the UTNGt21O strain has a particular gene composition with probiotic features, encoding for several proteins and enzymes responsible for the carbohydrate, nucleotide, and energy metabolism; metabolism of cofactors of vitamins and lipids; and secondary metabolite biosynthesis. The EggNOG annotation disclosed the presence of unique genes encoding for putative penicillin acetylase, riboflavin, and folate, suggesting its potential to produce proteins/enzymes with biotechnological significance, but these features must be proved in further *in vitro* studies. From the safety point of view, the genome analysis indicated that this strain is non-pathogenic, non-virulent, and has a stable genome as no mobile elements and acquired antibiotic resistance genes were detected. The presence of CRISPR-Cas systems might be used to design new strains with enhanced functional features. Pangenome analysis indicated the presence of strain-specific genes encoding for a putative RelB antitoxin, a putative pesticidal crystal protein Cry22Aa, a putative type-1 restriction enzyme R protein, and type-I restriction enzyme EcoKI M, responsible for the defense mechanism. Besides, *in vitro* molecular analysis confirmed the antimicrobial capacity of the UTNGt21O peptide extracts toward foodborne pathogens. Thus, a direct interaction between the peptide extracts and *S. aureus* cell results in cell integrity loss, the release of aromatic molecules, and possible breaks in the target membrane proteins, provoking cell death. Further *ex vitro* studies are required to confirm its overall inhibitory effectiveness in different foods. Nonetheless, these promising features retrieved from the genome sequencing analysis will concede for further developing novel bioproducts with *Weissella* species and their associated metabolites. This might provide improved bioactive and technological properties, as well as the design of novel inhibitory bio-formulations that may reduce the contamination by microorganisms of fresh tropical fruits, increasing their shelf-life from farm to perch, which would strengthen the market chains and allow the commercialization of good-quality, improved-aspect, and antimicrobial-protected products.

## Data Availability Statement

The datasets presented in this study can be found in online repositories. The names of the repository/repositories and accession number(s) can be found in the article/Supplementary Material.

## Author Contributions

Both authors contributed to the investigation and formal analysis. GT contributes to conceptualization, methodology, data curation, supervision, project administration, funding acquisition, writing—original draft preparation, writing—review, and editing. Both authors have read and agreed to the published version of the manuscript.

## Conflict of Interest

The authors declare that the research was conducted in the absence of any commercial or financial relationships that could be construed as a potential conflict of interest.

## Footnotes

1 http://www.cbs.dtu.dk/services/RNAmmer/

2 http://www.ansikte.se/ARAGORN/

3 http://www.cbs.dtu.dk/services/SignalP/

4 https://crisprcas.i2bc.paris-saclay.fr/CrisprCasFinder/Index

5 http://phaster.ca

6 http://arpcard.mcmaster.ca/

7 https://cge.cbs.dtu.dk/services/ResFinder/

8 https://cge.cbs.dtu.dk/services/PlasmidFinder-2.0/

9 http://www.mgc.ac.cn/VFs/main.htm

10 http://cge.cbs.dtu.dk/services/PathogenFinder/

11 https://www.ebi.ac.uk/interpro/search/sequence/

12 http://pfam.xfam.org

13 https://www.ncbi.nlm.nih.gov/cdd/

14 http://eggnog5.embl.de/#/app/home

15 https://www.uniprot.org/statistics/Swiss-Prot

16 https://metacyc.org

17 http://www.mgc.ac.cn/VFs/main.htm

18 http://www.phi-base.org/searchFacet.htm?queryTerm

19 http://xfam.org

20 http://gctype.wdcm.org

## Supplementary Material

Supplementary Figure 1 **(A)** Percent identity heatmap resulting from ANI analysis. **(B)** Alignment coverage heatmap resulting from ANI analysis.

Supplementary Figure 2 The protein alignment of the putative penicillin acylase of UTNGt21O (Query, black color) and penicillin V acylase of *Lysinibacillus sphaericus* (subject, red color).

Supplementary Figure 3 NCBI BLAST tree view based on the pairwise alignment of putative penicillin acylase protein sequence of UTNGt21O and the proteins from different taxa (NCBI PDB protein database).

Supplementary Table 1 Lists of strains used in the pangenome analysis.

Supplementary Table 2 BLAST results of top five contigs that match the UTNGt21O strain.

Supplementary Table 3 Gene prediction statistics.

Supplementary Table 4 Putative CRISPR-Cas detection with the CRISPRFinder web service.

Supplementary Table 5 Genes annotated with the CARD protein ID and grouped by drug class, resistance mechanism, and AMR gene family.

Supplementary Table 6 Antibiotic susceptibility of the UTNGt21O strain.

Supplementary Table 7 Description of putative genes predicted with the virulence factor database (VFDB).
