# Supplementary material for: Next-Generation Sequencing for Whole-Genome Characterization of Weissella cibaria UTNGt21O Strain Originated From Wild Solanum quitoense Lam. Fruits: An Atlas of Metabolites With Biotechnological Significance
Source: Front Microbiol. 2021 Jun 7;12:675002. doi: 10.3389/fmicb.2021.675002 (PMC8215347; doi:10.3389/fmicb.2021.675002)
Supplement: Supplementary Figure 1 — (A) Percent identity heatmap resulting from ANI analysis. (B) Alignment coverage heatmap resulting from ANI analysis. [file Data_Sheet_1.docx]

**Supplementary Figure S1.** Percent identity heatmap resulted by ANI analysis. The cells in the heatmap corresponding to an ANI value of 95% and higher are stained red. This indicates that the corresponding strains belong to the same species. The dendrograms (in green; above and on the left side), which were constructed by the simple linkage of the ANIm (ANI with MUMmer) percentage identities, correspond to the results of the clustering of the ANI values between the used strains (Pritchard et al., 2016). **B.** Alignment coverage heatmap resulted by ANI analysis. Heatmap of ANI coverage for 5 bacterial isolates as described in Table 1 and the Query UTNGt21O. The isolates and species assignments as indicated at source are given as row and column labels. Cells in the heatmap corresponding to 75% coverage or greater are colored red. Blue cells correspond to coverage of 50% or less. Color intensity fades as the comparisons approach 50% coverage. The dendrograms (in green; above and on the left side) of the heatmap correspond to strains assignments for each isolate in the analysis. ANI: Average Nucleotide Identity

A.

B.

**Supplementary Figure S2.** The protein alignment of the penicillin acylase of UTNGt21O (Query, black color) and Penicillin V Acylase of *Lysinibacillus sphaericus* (sbjct, red color)


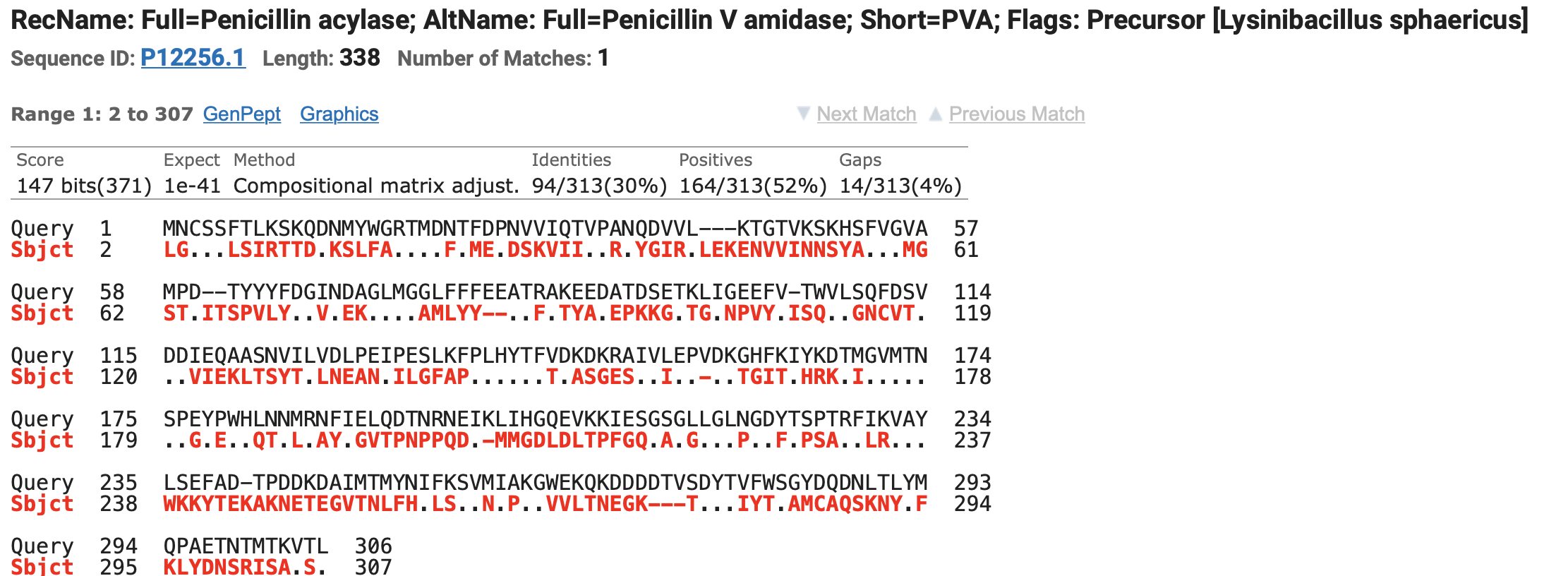


**Supplementary Figure S3.** NCBI Blast tree view based on the pairwise alignment of putative penicillin acylase protein sequence of UTNGt21O and the proteins from different taxa (NCBI PDB protein database).
