# Supplementary material for: Next-Generation Sequencing for Whole-Genome Characterization of Weissella cibaria UTNGt21O Strain Originated From Wild Solanum quitoense Lam. Fruits: An Atlas of Metabolites With Biotechnological Significance
Source: Front Microbiol. 2021 Jun 7;12:675002. doi: 10.3389/fmicb.2021.675002 (PMC8215347; doi:10.3389/fmicb.2021.675002)
Supplement: Supplementary Table 1 — Lists of strains used in the pangenome analysis. [file Data_Sheet_2.docx]

**Supplementary Table S1.** Lists of strains used in the pangenome analysis

| **Strains** | **NCBI Accession** |
| --- | --- |
| CP027427.1 *W. cibaria* strain BM2 chromosome, complete genome | [CP027427.1](http://www.ncbi.nlm.nih.gov/sites/entrez?db=nuccore&cmd=search&term=CP027427.1) |
| CP035267.1 *W. cibaria* strain SRCM103448 chromosome, complete genome | [CP035267.1](http://www.ncbi.nlm.nih.gov/sites/entrez?db=nuccore&cmd=search&term=CP035267.1) |
| CP022606.1 *W. cibaria* strain CMS1, complete genome | [CP022606.1](http://www.ncbi.nlm.nih.gov/sites/entrez?db=nuccore&cmd=search&term=CP022606.1) |
| CP013936.1 *W. cibaria* strain CMU, complete genome | [CP013936.1](http://www.ncbi.nlm.nih.gov/sites/entrez?db=nuccore&cmd=search&term=CP013936.1) |
| CP013726.1 *W. cibaria* strain CMS2, complete genome | [CP013726.1](http://www.ncbi.nlm.nih.gov/sites/entrez?db=nuccore&cmd=search&term=CP013726.1) |
| CP041193.1 *W. cibaria* strain CBA3612 chromosome, complete genome | [CP041193.1](http://www.ncbi.nlm.nih.gov/sites/entrez?db=nuccore&cmd=search&term=CP041193.1) |
| CP012873.1 *W. cibaria* strain CH2, complete genome | [CP012873.1](http://www.ncbi.nlm.nih.gov/sites/entrez?db=nuccore&cmd=search&term=CP012873.1) |
| CP024929.1 *Leuconostoc citreum* strain EFEL 2700 chromosome, complete genome | [CP024929.1](http://www.ncbi.nlm.nih.gov/sites/entrez?db=nuccore&cmd=search&term=CP024929.1) |
| CP023501.1 *W. paramesenteroides* strain FDAARGOS_414 chromosome, complete genome | [CP023501.1](http://www.ncbi.nlm.nih.gov/sites/entrez?db=nuccore&cmd=search&term=CP023501.1) |
| CP026847.1 *W. koreensis* strain WiKim0080 chromosome, complete genome | [CP026847.1](http://www.ncbi.nlm.nih.gov/sites/entrez?db=nuccore&cmd=search&term=CP026847.1) |

**Supplementary Table S2.** BLAST results of top five contigs that match the UTNGt21O strain

| **Contig** | **Contig Length** | **Subject Description** | **Subject Length** | **E-Value** |
| --- | --- | --- | --- | --- |
| Contig 1 | 422,230 | CP035267.1 *W. cibaria* strain SRCM103448 chromosome, complete genome | 2,397,676 | 0.0 |
| Contig 2 | 284,835 | CP027563.1 *W. confusa* strain VTT E-133279 chromosome, complete genome | 2,212,145 | 0.0 |
| Contig 3 | 203,430 | CP042399.1 *W. hellenica* strain CBA3632 chromosome, complete genome | 1,900,683 | 0.0 |
| Contig 4 | 141,980 | CP041193.1 *W. cibaria* strain CBA3612 chromosome, complete genome | 2,382,774 | 0.0 |
| Contig 5 | 132,017 | CP012873.1 *W. cibaria* strain CH2, complete genome | 2,466,961 | 0.0 |

Legend: Contig: The name of contig; Contig length (bp) : The total number of bases in the contig; Subject description : Description of sequence matched by BLASTN; Subject length (bp) : Length of the sequence matched by BLASTN; E-value : The expectations that could be matched by chance. The lower, the more significant it is.

**Supplementary Table S3.** Gene prediction statistics

| **Sample/ Number Contigs** | **Genome size (Kbp)** | **Number of genes** | **Total length (Kbp)** | **Cover (%)** | **Average length (bp)** | **Average intergenic (bp)** | **Partial gene ratio (%)** |
| --- | --- | --- | --- | --- | --- | --- | --- |
| UTNGt21O/ 31 | 1924.087 | 1867 | 1654.05 | 0.86 | 882.16 | 1035.95 | 0.02 |

**Supplementary Table S4.** Putative CRISPR-Cas detection with the CRISPRFinder webservice

| **Element** | **CRISPR ID/ Cas Type** | **Start** | **End** | **Spacer/ Gene** | **Repeat consensus/ Cas gene** | **Direction** | **Evidence level (number of repeats)** |
| --- | --- | --- | --- | --- | --- | --- | --- |
| CRISPR | CRISPR 1/ contig 5_1 | 121014 | 121114 | 1 | 5’-TGCTCTACCAACTGAGCTAAGTCGG-3’ | Non determined | 1 |
| Cas cluster | CAS putative/ contig 8 | 77077 | 78147 | 1 | Cas3_0_1 | Non determined | 0 |

**Supplementary Table S5**. Genes annotated with the CARD protein ID and grouped by Drug Class, Resistance Mechanism and AMR gene family. The number of genes in each category is shown.

| Genes | Number of genes | Cathegory |
| --- | --- | --- |
| PtsI phosphotransferase | 1 | AMR_Gene_Family |
| daptomycin resistant pgsA | 1 | AMR_Gene_Family |
| glycopeptide resistance gene cluster | 2 | AMR_Gene_Family |
| antibiotic resistant rpsL | 1 | AMR_Gene_Family |
| fluoroquinolone self-resistant parC | 1 | AMR_Gene_Family |
| ATP-binding cassette (ABC) antibiotic efflux pump | 2 | AMR_Gene_Family |
| tetracycline-resistant ribosomal protection protein | 1 | AMR_Gene_Family |
| Penicillin-binding protein mutations conferring resistance to beta-lactam antibiotics | 1 | AMR_Gene_Family |
| fluoroquinolone resistant gyrA | 1 | AMR_Gene_Family |
| daptomycin resistant cls | 2 | AMR_Gene_Family |
| resistance-nodulation-cell division (RND) antibiotic efflux pump | 1 | AMR_Gene_Family |
| antibiotic resistant isoleucyl-tRNA synthetase (ileS) | 1 | AMR_Gene_Family |
| ABC-F ATP-binding cassette ribosomal protection protein | 2 | AMR_Gene_Family |
| antibiotic resistant fusA | 1 | AMR_Gene_Family |
| aminosalicylate resistant thymidylate synthase | 1 | AMR_Gene_Family |
| aminocoumarin resistant parE | 1 | AMR_Gene_Family |
| aminocoumarin resistant gyrB | 1 | AMR_Gene_Family |
| daptomycin resistant beta-subunit of RNA polymerase (rpoB) | 1 | AMR_Gene_Family |
| murA transferase | 1 | AMR_Gene_Family |
| fluoroquinolone resistant parC | 1 | AMR_Gene_Family |
| rifamycin-resistant beta-subunit of RNA polymerase (rpoB) | 1 | AMR_Gene_Family |
| small multidrug resistance (SMR) antibiotic efflux pump | 2 | AMR_Gene_Family |
| major facilitator superfamily (MFS) antibiotic efflux pump | 5 | AMR_Gene_Family |
| vanR | 2 | AMR_Gene_Family |
| antibiotic resistance fabG | 1 | AMR_Gene_Family |
| daptomycin resistant CdsA | 1 | AMR_Gene_Family |
| antibiotic resistant fusE | 1 | AMR_Gene_Family |
| daptomycin resistant beta prime subunit of RNA polymerase (rpoC) | 1 | AMR_Gene_Family |
| elfamycin resistant EF-Tu | 1 | AMR_Gene_Family |
| Triclosan | 1 | Drug_Class |
| macrolide antibiotic | 1 | Drug_Class |
| rifamycin antibiotic | 2 | Drug_Class |
| acridine dye | 2 | Drug_Class |
| tetracycline antibiotic | 5 | Drug_Class |
| peptide antibiotic | 7 | Drug_Class |
| fosfomycin | 3 | Drug_Class |
| mupirocin | 1 | Drug_Class |
| lincosamide antibiotic | 3 | Drug_Class |
| glycopeptide antibiotic | 2 | Drug_Class |
| fluoroquinolone antibiotic | 4 | Drug_Class |
| nybomycin | 1 | Drug_Class |
| fusidic acid | 2 | Drug_Class |
| monobactam | 1 | Drug_Class |
| para-aminosalicylic acid | 1 | Drug_Class |
| carbapenem | 1 | Drug_Class |
| aminoglycoside antibiotic | 3 | Drug_Class |
| phenicol antibiotic | 2 | Drug_Class |
| cephalosporin | 1 | Drug_Class |
| streptogramin antibiotic | 1 | Drug_Class |
| cephamycin | 1 | Drug_Class |
| pleuromutilin antibiotic | 2 | Drug_Class |
| Penam | 2 | Drug_Class |
| elfamycin antibiotic | 1 | Drug_Class |
| aminocoumarin antibiotic | 2 | Drug_Class |
| antibiotic target protection | 3 | Resistance_Mechanism |
| antibiotic target alteration | 23 | Resistance_Mechanism |
| antibiotic target replacement | 1 | Resistance_Mechanism |
| antibiotic efflux | 10 | Resistance_Mechanism |

**Supplementary Table S6.** Antibiotic susceptibility of the UTNGt21O strain

| **Antimicrobial E-test strip** | **MIC* (mg/L)** | **Susceptibility**** | **EFSA cut-off values*** (mg/L) *Lactobacillus* obligate**  **Heterofermentative (EFSA, 2012)** |
| --- | --- | --- | --- |
| Amoxicillin | 0.25 | S | 4 |
| Amoxicillin: clavulanic acid | 0.125 | S | 4 |
| Ampicillin | 0.25 | S | 2 |
| Cefotaxime | 0.25 | S | 16 |
| Erythromycin | 2 | S | 1 |
| Gentamycin | 48 | R | 16 |
| Penicillin | 0.25 | S | 0.25 |
| Tetracycline | 2 | S | 8 |

* The microbiological breakpoints (MIC) reported by the FEEDAP were used to categorize lactobacilli as susceptible or resistant. **The strains showing a MIC higher than the EFSA breakpoint were considered resistant. Susceptible (S): a bacterial strain is defined as susceptible when it is inhibited at a concentration of a specific antimicrobial equal to or lower than the established cut-off value (S ≤ x mg / L). Resistant (R): a bacterial strain is defined as resistant when it is not inhibited at a concentration of a specific antimicrobial above the established cut-off value (R> x mg / L). *** No specific MIC breakpoint values for *Weissella* species have been defined by EFSA for the assessment of antimicrobial susceptibility, thus the cut-off values from *Lactobacillus* were considered for the results interpretation.

**Supplementary Table S7.** Description of putative genes predicted with the virulence factor database (VFDB).

| Query ID | % Identity | Description |
| --- | --- | --- |
| gene00026 | 71.5 | VFG006826(gi:16803417) (lisR) two-component response regulator |
| gene00045 | 40 | VFG036552(gi:385339601) (fbpC) iron (III) ABC transporter ATP-binding protein |
| gene00048 | 40.1 | VFG005382(gi:116628017) (srtA) Sortase (surface protein transpeptidase) |
| gene00080 | 44.8 | VFG005182(gi:94992312) (fbp54) Fibronectin-binding protein / Fibrinogen-binding protein |
| gene00124 | 46.4 | # |
| gene00263 | 44.6 | VFG046612 (FN3523_1292) Ribulose-phosphate 3-epimerase |
| gene00266 | 40.6 | VFG032386(gi:347549217) (stp) putative phosphoprotein phosphatase |
| gene00280 | 48.3 | # |
| gene00310 | 41.8 | VFG012939(gi:110804318) (gtrB) bactoprenol glucosyl transferase |
| gene00354 | 41.6 | VFG016229(gi:30020330) (hlyIII) Hemolysin III |
| gene00373 | 42.8 | VFG038840(gi:507521851) (flmH) 3-oxoacyl-ACP reductase |
| gene00379 | 41.5 | VFG011402(gi:23502030) (fabZ) (3R)-hydroxymyristoyl ACP dehydratase |
| gene00389 | 69 | VFG012101(gi:15895960) (groEL) chaperonin GroEL |
| gene00404 | 56 | VFG005353(gi:76787761) (plr/gapA) glyceraldehyde-3-phosphate dehydrogenase |
| gene00448 | 41.2 | VFG008680(gi:433650447) (ddrA) daunorubicin resistance ABC transporter ATP-binding subunit |
| gene00488 | 42.1 | VFG015379(gi:152986872) (PSPA7_0144) probable ATP-binding component of ABC transporter |
| gene00574 | 47.3 | VFG016424(gi:118480308) (manA) mannose-6-phosphate isomerase |
| gene00598 | 40.6 | VFG005382(gi:116628017) (srtA) Sortase (surface protein transpeptidase) |
| gene00603 | 56.2 | # |
| gene00626 | 57.2 | VFG016309(gi:49481245) (galE) UDP-glucose 4-epimerase |
| gene00712 | 42.8 | VFG043456(gi:22537734) (scpB) segregation and condensation protein B |
| gene00755 | 60.3 | VFG043573(gi:15605121) (CT396) molecular chaperone DnaK |
| gene00764 | 48.9 | VFG049038 (KPN2242_15480) hypothetical protein |
| gene00766 | 73.1 | # |
| gene00768 | 53.2 | VFG005986(gi:55823035) (epsE) exopolysaccharide biosynthesis protein |
| gene00824 | 47.5 | VFG031486(gi:379746526) (ndk) nucleoside diphosphate kinase |
| gene00856 | 45.7 | VFG019071(gi:169832565) (lytA) lytic amidase (N-acetylmuramoyl-L-alanine amidase) |
| gene00889 | 40.2 | VFG026433(gi:383308019) (glnA1) glutamine synthetase |
| gene00940 | 41.4 | # |
| gene00944 | 44.1 | # |
| gene00972 | 43.9 | VFG016506(gi:71894295) (pdhB) pyruvate dehydrogenase E1 component, beta subunit |
| gene00974 | 40.3 | VFG032200(gi:347548334) (lplA1) putative lipoate protein ligase A |
| gene00977 | 44.9 | VFG024188(gi:406029299) (mprA) response regulator mprA |
| gene00994 | 46 | VFG013269(gi:33152420) (orfM) putative deoxyribonucleotide triphosphate pyrophosphatase |
| gene01003 | 62.6 | VFG005552(gi:125718786) (tig/ropA) Trigger factor, putative |
| gene01005 | 73.8 | VFG016490(gi:42560712) (tuf) translation elongation factor Tu |
| gene01078 | 44.9 | VFG019089(gi:169832575) (htrA/degP) trypsin domain protein |
| gene01083 | 49.6 | VFG031738(gi:169631126) (regX3) Sensory transduction protein RegX3 |
| gene01088 | 46.8 | VFG006719(gi:16800743) (lap) hypothetical protein |
| gene01138 | 66.7 | VFG048845 (KOX_25145) 6-phosphogluconate dehydrogenase |
| gene01155 | 61.3 | VFG026980(gi:386005579) (sigA/rpoV) RNA polymerase sigma factor |
| gene01156 | 44 | VFG031738(gi:169631126) (regX3) Sensory transduction protein RegX3 |
| gene01190 | 41.8 | VFG032788(gi:16800042) (dltA) D-alanine--poly(phosphoribitol) ligase subunit 1 |
| gene01205 | 40.7 | VFG047569 (FNFX1_0412) hypothetical protein |
| gene01249 | 48.6 | VFG005372(gi:116516991) (slrA) peptidyl-prolyl cis-trans isomerase, cyclophilin-type |
| gene01256 | 51.6 | VFG012177(gi:18310800) (hlyD) probable hemolysin |
| gene01289 | 59.4 | # |
| gene01314 | 60.9 | # |
| gene01350 | 43.5 | VFG013515(gi:148826007) (mrsA/glmM) predicted phosphomannomutase |
| gene01365 | 69.2 | VFG005580(gi:15900994) (eno) phosphopyruvate hydratase |
| gene01372 | 71.4 | VFG005865(gi:24378821) (SMU.322c) glucose-1-phosphate uridylyltransferase |
| gene01379 | 52.6 | VFG005372(gi:116516991) (slrA) peptidyl-prolyl cis-trans isomerase, cyclophilin-type |
| gene01383 | 44.2 | VFG030696(gi:387874659) (sugC) ABC transporter, ATP-binding protein SugC |
| gene01397 | 40.5 | VFG031747(gi:406032839) (regX3) Sensory transduction protein regX3 |
| gene01409 | 47.9 | VFG022910(gi:333992254) (phoP) two-component system response phosphate regulon transcriptional regulator PhoP |
| gene01426 | 47.9 | VFG015903(gi:71735587) (argK) phaseolotoxin-insensitive ornithine carbamoyltransferase |
| gene01459 | 56.1 | VFG019078(gi:182684949) (plr/gapA) glyceraldehyde-3-phosphate dehydrogenase |
| gene01460 | 54.1 | VFG005582(gi:125717729) (eno) Enolase, putative |
| gene01644 | 62.5 | VFG045688(gi:383329042) (uppS) undecaprenyl diphosphate synthase |
| gene01645 | 49.4 | # |
| gene01654 | 50.1 | VFG037029(gi:59802086) (katA) catalase |
| gene01815 | 54.2 | VFG005579(gi:116516768) (eno) phosphopyruvate hydratase |
